# Financial crisis and income-related inequalities in the universal provision of a public service: the case of healthcare in Spain

**DOI:** 10.1186/s12939-017-0630-y

**Published:** 2017-07-24

**Authors:** Ignacio Abásolo, Marc Saez, Guillem López-Casasnovas

**Affiliations:** 1 Department of Applied Economics and Quantitative Methods, University of La Laguna, La Laguna, Spain; 2 University Institute of Regional Development, University of La Laguna, La Laguna, Spain; 3 Research Group on Statistics, Econometrics and Health (GRECS) University of Girona, Carrer de la Universitat de Girona 10 Campus de Montilivi, 17003 Girona, Spain; 4 CIBER of Epidemiology and Public Health (CIBERESP), Madrid, Spain; 5 Center for Research in Health and Economics (CRES), University Pompeu Fabra, Barcelona, Spain; 6 Department of Economics and Business, University Pompeu Fabra, Barcelona, Spain; 7 Barcelona Graduate School (BSGE), University Pompeu Fabra, Barcelona, Spain

**Keywords:** Financial crisis, Universality, Health services, Inequality, Access to the health system

## Abstract

**Background:**

The objective of this paper is to analyse whether the recent recession has altered health care utilisation patterns of different income groups in Spain.

**Methods:**

Based on information concerning individuals ‘income and health care use, along with health need indicators and demographic characteristics (provided by the Spanish National Health Surveys from 2006/07 and 2011/12), econometric models are estimated in two parts (mixed logistic regressions and truncated negative binominal regressions) for each of the public health services studied (family doctor appointments, appointments with specialists, hospitalisations, emergencies and prescription drug use).

**Results:**

The results show that the principle of universal access to public health provision does not in fact prevent a financial crisis from affecting certain income groups more than others in their utilisation of public health services.

**Conclusions:**

Specifically, in relative terms the recession has been more detrimental to low-income groups in the cases of specialist appointments and hospitalisations, whereas it has worked to their advantage in the cases of emergency services and family doctor appointments.

## Background

Universality in a public service like health care is usually identified in its aspects relative to access to the service, irrespective of individual financial means. If the distribution of relative need was equal and there was propensity to treat all groups identically, universalism would generate a utilisation that was proportional to the weight of the population in each stratum analysed. The real world is, of course, much more complex and debate is called for as to how to implement universality when, particularly during a crisis, in countries like Spain where the distribution of relative need is unequal [1], income related health inequalities are present, and social policy is not always sensitive to results the impacts on equity [2]. Utilisation is may then be the leading factor for the sign of final incidence on income redistribution (pro-poor, pro-rich) that a universal health system may create.

The effects of the past/current crisis on health systems as reported in *Health policy responses to the financial crisis in Europe,* demonstrates the diverse responses to the recession [3]. Our objective is to analyse whether the crisis has altered the patterns of how income groups use the principal public health services and the subsequent consequences.

The following are some of the questions that prompted our study: What effect do the marginal public health service reductions (brought about by the recession) have on income groups (income quartiles)? Given the changing pattern of utilisation for each of the public services analysed, was a regressive or a progressive effect introduced after the onset of the crisis? When analysing possible explanations, are low-income groups more adversely affected because the rich have abandoned double medical cover and/or private utilisation and opted to join a public health service left with less resources? Or, in light of the deterioration of the public health service, has the number of individuals in the lower and medium-income quartiles with private health insurance increased? Or the reverse: have high income groups abandoned scraped public services, increasing private insurance, and in so doing relatively benefitted lower-income quartiles who are mostly users? Obviously, whatever the effect may be, the result will depend largely on individual preferences not only because public/private quality ‘as perceived by the patient’ can vary from one service to another, but also because user access to the various services differs (primary healthcare appointments and emergencies, which are initiated by the patient, as opposed to specialist health appointments and hospital care, which is ordered by the supplier).

Note that our aim is to analyse the redistributive effect the crisis has had as a result of the changes in public health utilisation by income groups. Until now, practically all the literature has been concerned with the combined effect not only of income, but also of other socioeconomic variables (education, occupation, work situation, etc.). In doing so - apart from possible problems of multicollinearity between these variables - the redistributive effect (income effect) can have become obscured or blurred without being able to be measured.

We control here for different need factors that are supposed to we assume could influence public health service utilisation: Has the demographic composition of the income quartiles (age/sex) changed? Furthermore, has the approximate distribution of mental health needs changed for income quartiles? Has there been any significant change in the demand for private health insurance since the crisis began? By pursuing these effects, not only do we consider the probability of using the different health services, but we also include frequentation in the analysis of the different health services. In particular, we analyse, separately, those public health services where, in the existing literature to date, frequentation has not been analysed.

In the first section, we survey what we do and do not know on the topic, in the second section we proceed to build our empirical estimation and in the third we show our results and discuss the evidence raised from the Spanish experience.

### What we do know about the redistributive effects of the Spanish health system

Most of the studies on the incidence of the Spanish public social expenditure by income levels for the decade of the 1980s highlighted that given that health care has been universal since 1986, the redistributive impact of public spending on household expenditure is given, above all, by direct assistance is much more. However, Calonge and Manresa [4] were the first to demonstrate that the use observed itself showed a limited contribution to redistribution. This means that public health utilisation among the income deciles varied very little in absolute terms after universal access, although the results obtained by Ortiz et al. for the period 1993–94 indicate a higher relative concentration of public spending on health by the intermediate grades of income distribution [5].

Amongst the more recent related research, Calero and Gil [6] showed that despite public health spending being progressive in its calculations (in absolute terms, with a concentration index of −0.1048) and redistributive in its effects (meaning that it complies with the aims of equity which, a priori, are attributed to health policy), its progressivity and redistribution indexes for 2010 were worse than for 2005. This is the case even though its relative redistributive role was more important, since it allowed for a greater reduction in inequality in 2010 than in 2005. In 2010, health spending largely benefitted the least advantaged social groups and, to some degree, the middle classes. In comparison to 2005, this spending did not carry as much weight in relation to income for the poorest households, while for wealthier households the percentage was higher. At any rate, Calero and Gil concluded that the biggest contributor to progressivity was primary healthcare expenditure, followed by the cost of emergencies. In general, both the starting point (initial income) and the final position (disposable income) was worse overall in 2010 that in 2005 in terms of income inequality. In both years, public health spending contributed greatly to easing this overall worsening of income inequality, although a comparison with previous decades shows a return to 1990s values in terms of both progressivity and redistribution.

Most of the studies have found that health service utilisation by different socioeconomic levels (income level, social class, education, etc.) is the leading factor for those differential impacts. Evidence to shows that primary healthcare tends to be pro-poor and specialist healthcare pro-rich (although results for hospitalisations have been less conclusive), especially if adjustments are made for ‘health need’ (see Urbanos et al. [7], Regidor et al. [8, 9], González and Clavero [10] and Abásolo et al. [11, 12] for Spain, Morris et al. [13] and Cookson et al. [14] for the United Kingdom and Agerholm et al. for Sweden [15]).

The question is still an open debate. External shocks change utilisation, as do relative needs and the responses the universal health system gives. For instance, in a study for the period 1987–2001, it was shown that in Spain income differences for people with the same level of need did not lead to differences in access to healthcare (medical consultations, emergency treatment and hospitalisations) [9]. On the contrary, Abásolo et al. [12] showed that in general there is no equity in access to the public health system services by different socioeconomic levels [10]. They reached this conclusion when the two aspects of public health system acess - utilisation and waiting times - were considered simultaneously, a methodology that appears to be suitable once it was shown that those who use the different health services analysed have different characteristics from those who do not use it (i.e. a problem of selection bias).

In brief, for the most recent period, some conjectures concerning utilisation changes may emerge from the impact generated by the economic recession. If private health spending is elastic with respect to income, a likely effect is that this would mainly affect the lowest income deciles. On the contrary, price and increased restriction to access should change the utilisation of the primary health care services (the part of the system most favourable to the poor in terms of its impact on the redistribution of income) of lower income groups. A first reading of the data points towards this. Between 2006 and 2011 primary health care utilisation went down in Spain by around 20–30%, especially among lower income groups who wished to avoid putting their jobs at risk by absenteeism (with family doctors signing them off sick). Additionally, the greater importance given in the Spanish health system to the role of nurses for pluripathology and chronic conditions and the greater amount of treatments outside the health centres, as well as innovations like electronic prescriptions, may have also served to reduce the number of appointments. In these last cases, lower utilisation would not necessarily put health at risk but rather reduce the redistribution impacts in the way we estimate at present. On the opposite side, hospital care (which is predominantly pro-rich) shows a more permanent frequentation and, therefore, it seems it has been less affected by the crisis, making for less favourable pro-poor relative redistributive impacts.

Following some of these conjectures, we explore the redistributive effects of an exogenous shock - the financial crisis - on the Spanish universal health system in a more systematic way. We do this by estimating what the impacts of changes in utilisation for income groups on different components of public health care expenditure are (family doctor appointments, engagements with specialists, hospitalisations, emergencies and medication use), and we discuss the causes and consequences for each of them.

## Methods

In short, in this paper we explore the redistributive effects of an exogenous shock - the financial crisis - on a universal health provision system. We want to track the consequences of the recession and austerity on health care utilisation. We do this by estimating the impacts of changes in utilisation for income groups on different components of public heath care expenditure (family doctor appointments, engagements with specialists, hospitalisations, emergencies and medication use), and for each of them we discuss their causes and consequences in the redistribution targets of the Spanish universal health care system.

### Data setting

We used microdata from the face-to-face cross-sectional population-based Spanish National Health Survey (SNHS) for two periods: 2006 (prior to the financial crisis) and 2011–2012 (during the financial crisis) [16, 17]. In this paper, we included adult individuals (aged 15 years or older) interviewed in both the adult and household questionnaires (*n* = 29,712 for 2006 and *n* = 19,935 for 2011–2012).

### Specification of the models

The response variables were the use of health care services - i) primary care, ii) specialized care, iii) hospitalisations and iv) emergency - as well as drugs consumption. All the response variables were counting variables: the number of visits in 1 year to primary and specialized health care services and to emergency services, the number of hospitalizations in 1 year, and the number of (different) medicines used in 1 year. Except in the case of drugs and medicines, we considered not only the use of health services in general, but we also distinguished between public and private ownership of these services (henceforth, total, public and private, respectively).

To meet our objective, (i.e. to analyse whether the crisis has altered the income groups’ utilization patterns of the main public health services and its consequences), for each sample (2006 and 2011–2012) we estimated three sets of models (one set for total, another for public and yet another for private). Each set contained three models, differentiated by the explanatory variables they contain (Table 1).

**Table 1:** Explanatory variables contained in the estimated models

Explanatory variables	Model 1	Model 2	Model 3
Net income of the individual’s family	X	X	X
Gender		X	X
Age		X	X
Private health insurance			X
Goldberg’s mental health index GHQ-12			X
Dependent variables			
Ownership of the health care services			
	Public	Private	
Primary care	X	X	
Specialized care	X	X	
Hospitalisations	X	X	
Emergency	X	X	
Drug and medicine consumption		X	

The first model in the set that only contained our main explanatory variable: the net income of the individual’s family (income from now on). We categorized income in quartiles, taking the first quartile as the reference category. The other two models include additional explanatory variables, with the idea of adjusting for the effects of income on the utilization. Thus, our second model contained, besides income, gender (male or female, with male as the reference category) and age. We categorized age as ‘15–35 years’, ‘36–45 years’, ‘46–55 years’, ‘56–65 years’, ‘66–75 years’, ‘75 years or older’, taking ‘15–35 years’ as the reference. Finally, in the third model, we included, along with income, sex and age, an indicator of having private health insurance (1 private health insurance, 0 other case) and the Goldberg’s mental health index GHQ-12 [18] (a counting variable, ranging 0–12).

### Estimation of the number of contacts and differentiating between publicly and privately-owned health services

The number of times that the individual has contacted health services in primary (‘family doctor’ in SNHS’ terms) and specialized care, as well as hospitalizations, is asked directly by the SNHS. In the case of emergency services, the SNHS not only asks directly, but also indirectly via other related questions, in particular those concerning hospitalization.

However, with the information provided by the health surveys, it was not possible to discern the number of contacts with a publicly or privately-owned health service. To estimate such numbers, we used information in relation to the last contact with the health service (also contained in the health surveys). All the analyses were done separately for SNHS-2006 and for SNHS-2011-2012.

First, we identified those individuals who reported having used the service only once.^1^

Next, we built an indicator of the ownership of the service of this single visit (1 private, 0 public). To do this, we used the questions, ‘Was the doctor you went to for your last consultation in private healthcare/otherwise?’, and, ‘Where did your last consultation take place (private health consultation/otherwise)?’, for primary and specialized health care, whereas for hospitalizations we used, ‘The last time you used an emergency department, what type of service did you use?’ (private healthcare/otherwise) and, ‘Who paid for the expenses of your last hospitalization?’ (private healthcare/otherwise).

Using these indicators as dependent variables, we specified four (mixed) logistic regression models: one for primary health care, one for specialized health care, one for emergency services and one for hospitalizations, with the objective of estimating the probability that the corresponding health service used was private.

In these models, we included, as explanatory variables, sex, age (categorized as above), self-perceived health status (categorized as ‘very good’, ‘good’, ‘fair’, ‘bad’ and ‘very bad’, with ‘very good’ being the reference category) and the number of chronic health conditions. In this case, individuals declare whether they are suffering from this condition and/or if the doctor has diagnosed this condition. We chose the latter to build our variable, adding the positive responses.

Then, we used the estimates of the coefficients of these models to predict the number of contacts with privately owned health services for those individuals who reported having more than one contact with health services. Finally, we calculated the number of contacts with public owned health services, by subtracting the number of ‘private’ contacts from the ‘total’ number of contacts.

### Extrapolation to a year of the number of contacts

In the case of hospitalizations and emergency services, the SNHS asks about the number of contacts in a year (i.e. the last 12 months), while in the case of primary care and specialized care the SNHS asks for the number of contacts in the last 4 weeks. We opted to extrapolate to a year the number of (total, public and private) contacts with primary and specialized health care services (simply multiplying by 12), although only for those individuals who reported having at least one contact (in last 4 weeks) with these services.

However, extrapolation was not as simple for individuals who declared that they had had no contact with health services in the past 4 weeks. In fact, it may be that the time between the individual feeling the need (‘problem, discomfort or illness’, in SNHS terms) to contact a (primary or specialized) health service and the effective contact was greater than 4 weeks.

For this reason, using only those individuals who reported having used the (primary or specialized) service at least once, we estimated (mixed) logistic regression models. The dependent variable was an indicator of having sought a medical consultation more than 4 weeks after the individual perceived any problem, discomfort or illness, and the explanatory variables were the same as those listed above. Again, analyses were also done separately for SNHS-2006 and for SNHS-2011-2012.

In this case, we used the estimated coefficients to predict the probability that an individual, who declares not having contacted health services in the past 4 weeks, would contact them anyway. Specifically, we assumed a (single) contact with health services if the probability was greater than 0.5 and no contact otherwise.

### Construction of the variable number of drugs and medicines used

In the case of drug and medicine use, the SNHS provides a list of 22 medications and the individual has to indicate whether or not they have consumed them in the last 12 months. It is important to note that the SNHS asks whether any of them have been consumed, but does not ask about the amount consumed. We added the positive responses, excluding contraceptives, to build the variable.

### Standardization of the net family income between the two editions of the SNHS survey

The SNHS-2006 and SNHS-2011-2012 surveys contain questions about the net income of the individual’s family. First, the income to which the SNHS-2006 refers to is annual, while in SNHS-2011-2012 it is monthly. Second, the number of intervals for income is different for each survey (eight intervals in SNHS-2006, whereas there are ten in SNHS-2011-2012). Finally, the range for these intervals is also different (even when those in SNHS-2011-2012 are annualized), i.e. from ‘less than €350/month’ to ‘over €6000/month’ in SNHS-2006 and from ‘€550 or less/month’ to ‘over €3450/month’ in SNHS-2011-2012.

For these reasons, we decided to standardize the net family income. For that, we specified two (mixed) ordered probit models (one for SNHS-2006 and the other for SNHS-2011-2012), with the dependent variable being the net family income and with explanatory variables, sex, age (categorized as above), occupation (categorized as ‘working’, ‘unemployed less than a year’, ‘unemployed over a year’, ‘dedicated mainly to housework’, ‘student, ‘retiree or pensioner’, and ‘other’, taking ‘working’ as the reference category), studies (‘insufficient instruction’ - that is illiterate, uneducated or incomplete primary -, ‘primary studies’, ‘secondary studies’, ‘university studies’; taking ‘insufficient instruction’ as the reference category), and the number of family members.

We used the probability estimated in these models (again, separately for SNHS-2006 and for SNHS-2011-2012) as our (standardized) income variable. Finally, we categorize this new variable into quartiles.

### Estimating health service use

To meet our objective, we finished by estimating a two-part econometric model, a so-called ‘hurdle’ model, specified in such a way as to gather together the two decision processes theoretically involved in the use of medical care [19–21].

The first part of this decision process, that of seeking care (process performed by the subject), was modelled using a (mixed) logistic regression:

$$ \begin{array}{l}{\mu}_{1i}=\mathrm{Prob}\left({y}_i=1{\left|X\right.}_{1i},{\beta}_1\right)\\ {} \log \left(\frac{\mu_{1i}}{1-{\mu}_{1i}}\right)={{X^{\hbox{'}}}_1}_i{\beta}_1\\ {}Var\left({y}_{1i}{\left|X\right.}_{1i},{\beta}_1\right)={\mu}_{1i}\left(1-{\mu}_{1i}\right)\end{array} $$

where y denoted the variable response, X a matrix of explanatory variables (containing the intercept), β was the associated vector of unknown parameters and the subindices i and 1 denoted the individual and the first part of the decision in this case.

Please note that the real variance of the variable response was assumed to coincide with the theoretical, which is to say that the dispersion parameter was equal to the unit. This was so because the information available in this first part did not allow for simultaneous identification of the parameters associated to the conditional expectation or the parameters associated to the variance [19, 21].

In the second part of the model, the frequency of contacts (mainly determined by the doctor), the distribution of use (conditional to some use) was a (mixed) truncated negative binomial [19, 20].

$$ {f}_2\left({y}_i{\left|{X}_{2i},y\right.}_i>0,{\beta}_2\right)=\frac{\frac{\varGamma \left({y}_i+{\varPsi}_{2i}\right)}{\varGamma \left({\psi}_{2i}\right)\varGamma \left({y}_i+1\right)}{\left(\frac{\mu_{2i}}{\mu_{2i}+{\psi}_{2i}}\right)}^{y_i}}{{\left(\frac{\mu_{2i}+{\psi}_{2i}}{\psi_{2i}}\right)}^{\psi_{2i}}-1} $$

where Γ(.) was the gamma function: ${\mu}_{2i}= \exp \left({X}_{2i}^{\hbox{'}}{\beta}_2\right)$; ${\psi}_{2i}=\frac{\mu_{2i}}{\phi }$, *ϕ* was a dispersion parameter and the sub-index 2 denoted the second part of the decision process.

Note that we allowed both variables and, above all, the corresponding parameters in the first and second part to differ. The two parts of the model entered the likelihood function multiplicatively. It is important to note that we estimated the two parts simultaneously.

Above, we have used the word ‘mixed’ several times. This is because in all models we included random effects to capture the individual heterogeneity. We assumed they were identical and independent Gaussian random variables with constant variance, i.e. *υ*_*jt*_ ∼ *N*(0, *σ*_*υ*2_)

### Inference

Because of the relative complexity of our models, inferences were performed using a Bayesian framework. This approach is considered the most suitable in accounting for model uncertainty [22], particularly that which is associated with the estimation of our main explanatory variable income and the existence of individual heterogeneity. Furthermore, within the Bayesian approach it is easy to specify a hierarchical structure on the (observable) data and (unobservable) parameters, all considered random quantities. It is important to note this fact because it implies that, even when the random effects and regressors were correlated, estimators are consistent [23]. Within the (pure) Bayesian framework, we follow the Integrated Nested Laplace Approximation (INLA) approach [24, 25].

We used penalising complexity (PC) priors. These priors are invariant to re-parameterisations and have robustness properties [26].

All analyses were made with the free software R (version 3.2.3) [27], through the INLA package [28, 24].

## Results

Table 2 shows the descriptive statistics of the variables used in this research for both 2006 and 2011 with the mean difference and their statistical significance. The main changes in utilisation of the different public healthcare services between 2006 and 2011 illustrated by these descriptions are as follows: Appointments with public service specialists increased considerably (multiplying by 1.5), while appointments with public service family doctors and hospitalisations reduced by 13% and 11% respectively. Utilisation of public emergency services remained constant and medication use reduced by 8% after the onset of the crisis [5].

**Table 2:** Descriptive statistics

Variable	Definition	2006			2011			Difference	*p*-value^*^
		N	Mean	Std. Dev.	N	Mean	Std. Dev.		
APPUB	Use of primary health care public services	29,252	0.411	0.782	19,782	0.355	0.695	−13.7%	0.000
AEPUB	Use of specialist medical public services	29,252	0.189	0.596	7435	0.487	0.847	158.2%	0.000
HOSPPUB	Use of public health services, hospitalisations	28,905	0.106	0.386	18,865	0.094	0.415	−11.3%	0.000
URGPUB	Use of public health services, emergency services	28,227	0.426	1.493	19,212	0.428	1.051	0.6%	0.208
MEDICINES	Number of drugs consumed in the last 12 months	29,252	1.811	1.928	19,782	1.652	2.007	−8.8%	0.000
APPRI	Use of primary health care private services	29,252	0.069	0.131	19,782	0.045	0.089	−33.9%	0.000
AEPRI	Use of specialist medical private services	29,252	0.032	0.100	7435	0.062	0.109	97.7%	0.000
HOSPPRI	Use of private health services, hospitalisations	28,905	0.011	0.112	18,865	0.005	0.073	−57.3%	0.000
URGPRI	Use of private health services, emergency services	28,227	0.029	0.218	18,044	0.012	0.125	−58.3%	0.000
SEGUROP	private insurance (1 if yes; 0 otherwise)	29,252	0.125		19,281	0.134		7.2%	0.004
SEXO	gender (1 if female; 0 if male)	29,252	0.606		19,281	0.539		−11.2%	0.000
EDAD_15_35	age group (1 if age between 15 and 35; 0 otherwise)^a^	29,712	0.240		19,935	0.229		−4.6%	0.005
EDAD_36_45	age group (1 if age between 36 and 45; 0 otherwise)	29,712	0.198		19,935	0.187		−5.6%	0.002
EDAD_46_55	age group (1 if age between 46 and 55; 0 otherwise)	29,712	0.159		19,935	0.163		2.7%	0.206
EDAD_56_65	age group (1 if age between 56 and 65; 0 otherwise)	29,712	0.136		19,935	0.107		−21.3%	0.000
EDAD_66_75	age group (1 if age between 66 and 75; 0 otherwise)	29,712	0.134		19,935	0.136		1.3%	0.568
EDAD_75_MORE	age group (1 if older than 75; 0 otherwise)	29,712	0.118		19,935	0.145		23.6%	0.000
SMENTAL	mental health index	28,027	1.590	2.606	18,989	1.573	2.746	−1.1%	0.000

^*^ *p*-value for two-sample Wilcoxon rank-sum (Mann-Whitney) test for equality of medians in continuous variab or Fisher exact test for equality of proportions in categorical variables

^a^16 years for ENS 2006

When we stratify this information by income quartiles (see Table 3), it can be seen that the number of consultations with public specialists increased for all quartiles, but with varying intensity: there is a noticeably greater increase in utilisation among the highest quartiles, the 3rd and 4th (with 1.7 and 2.6 times more appointments after the onset of the crisis in 2006) than in the lowest quartiles, the 1st and 2nd (with 1.6 and 0.8 times more appointments), which leads us to conceive of a relative worsening in equity. As for appointments to see the family doctor, the decrease in frequentation for all quartiles is not homogenous either because while in the 1st quartile the number of appointments reduced by 38%, in the other quartiles this figure oscillated between 0.5% and 4%. For hospitalisations, the situation was similar as the only significant change happened for the 1st quartile, where utilisation practically disappeared. All in all, this points to a relative worsening of equity in terms of healthcare utilisation. The only public health service where the onset of the crisis would seem to be associated with a pro-poor effect has been the emergency services, as while individuals in the 4th quartile used this service 9.5% less, utilisation by individuals in the 1st quartile rose by 6.5% (utilisation for the other two quartiles was not affected). Lastly, in terms of medication use it seems that the crisis has favoured middle-income groups, amongst whom use has increased (while for the 4th quartile use decreased by 6.4% and, most notably, for the 1st quartile use decreased by almost half). This descriptive analysis indicates, therefore, that since the crisis began utilisation of public specialist healthcare services has increased, whereas utilisation of public primary healthcare and hospital services has decreased, which has favoured high-income groups. Meanwhile the change in the pattern of the public emergency services has been to the relative advantage of the lower-income groups. Medication use, on the other hand, has favoured middle-income groups rather than either high- or low-income groups.

**Table 3:** Health care utilization by income quartiles

	QUARTILE 1				QUARTILE 2				QUARTILE 3				QUARTILE 4			
	Means				Means				Means				Means			
	2006	2011	Diff.	*p*-value	2006	2011	Diff.	*p*-value	2006	2011	Diff.	*p*-value	2006	2011	Diff.	*p*-value
APPUB	0.544	0.342	−37.1%	0.000	0.506	0.503	−0.5%	0.000	0.333	0.320	−3.8%	0.000	0.262	0.251	−4.1%	0.019
AEPUB	0.195	0.514	163.3%	0.000	0.218	0.398	82.7%	0.000	0.175	0.482	176.2%	0.000	0.167	0.607	264.3%	0.000
HOSPUB	0.113	0.000	−99.8%	0.000	0.141	0.164	16.3%	0.667	0.090	0.114	26.5%	0.042	0.080	0.085	5.5%	0.666
URGPUB	0.353	0.376	6.5%	0.006	0.464	0.496	6.8%	0.618	0.471	0.472	0.3%	0.216	0.404	0.366	−9.5%	0.004
MEDICINES	2.620	1.440	−45.0%	0.000	2.299	2.610	13.5%	0.000	1.242	1.526	22.8%	0.000	1.092	1.022	−6.4%	0.000
APPRIV	0.044	0.032	−26.6%	0.000	0.056	0.044	−21.3%	0.000	0.085	0.041	−51.5%	0.000	0.091	0.065	−29.2%	0.000
AEPRIV	0.033	0.051	56.3%	0.000	0.028	0.062	121.7%	0.000	0.029	0.066	125.4%	0.000	0.037	0.078	113.1%	0.000
HOSPRIV	0.015	0.000	−100.0%	0.000	0.014	0.011	−20.3%	0.129	0.007	0.004	−39.6%	0.077	0.008	0.003	−68.0%	0.000
URGPRIV	0.021	0.000	−100.0%	0.000	0.037	0.028	−25.8%	0.003	0.031	0.012	−60.4%	0.000	0.026	0.005	−80.5%	0.000
SEGUROPRIV	0.100	0.155	54.7%	0.000	0.068	0.064	−6.7%	0.335	0.125	0.108	−13.2%	0.006	0.210	0.213	1.4%	0.700

It may be that part of the effects of the financial crisis can be attributable to changes - by income groups - in health needs (approximated by age, gender and people with mental health problems). In fact, Table 2 already indicates that the structure of age, sex and percentage of people with mental health problems has changed since the onset of the crisis (a change which, as we have already mentioned, could also be different by income quartiles). On the other hand, a variable that seems to be crucial in understanding the change in the patterns of public health service utilisation by levels of income, is whether an individual has double health cover or not (and if this has also changed for income groups).

Therefore, we have controlled for each of these variables in the estimation of different two-part regression models (termed a ‘*hurdle model’*), one for each type of health service and for each year, 2006 and 2011. Furthermore, this has been done in a sequential way: first, taking only the income quartiles into account and then adding the age and sex variables into the models, and finally adding the mental health and double medical cover variables. The results of this last, more comprehensive, model are shown in Table 4 (the other estimations are available upon request), both because it is the best model in terms of goodness of fit and because it is the one that best allows for the study of inequalities in utilisation which, a priori, are avoidable and unfair (i.e. differences in individual utilisation adjusted for health need – even though there could be other legitimate sources of inequality, such as those that derive from lifestyles, which we do not deal with in this research). It must also be pointed out that it is not necessarily true that more health service utilisation is better [29], but that more relative use by one (or some) specific income groups does imply more relative benefits of the resources destined for health, given that the financing system for many specific resources, in any case, is a symptom of the unequal utilisation of a universal service like the public health service, which is the object of the analysis of this research. The estimations indicate whether or not there is any significant difference by income quartiles in the likelihood of using the different health services (adjusted for health need and having double cover or not) and, especially, whether this has changed since the crisis began.

**Table 4:**
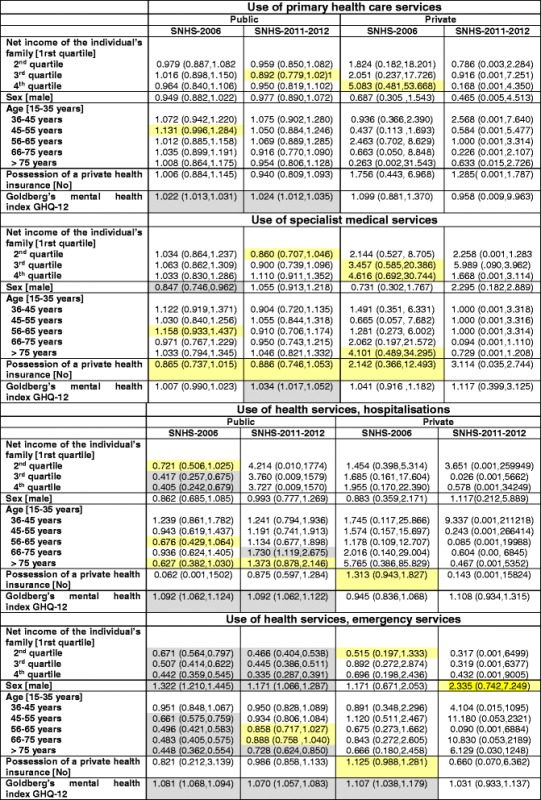
Results of the estimation of the hurdle models

Second part of the two-part econometric models

## Discussion

### Primary health care services

The results for 2006 for publicly funded primary health care show that once adjustment has been made for need (age, sex and state of mental health) and double health provision, there is no significant difference between income levels in the likelihood of using primary health services. This result is similar to the findings of studies that have made international comparisons [1, 30, 31], which have found little or no evidence of pro-poor inequity in family doctor appointments. The fact that these studies consider the total number of appointments (both public and private) must be taken into account, as this could curb the greater tendency among low-income groups to go to their family doctor, as observed in other studies that only consider publicly financed appointments. Some of these studies have been carried out in Spain [8–11], (involving, in particular, individuals over 50 years of age [32]), as well as in the UK [13] and Norway [33]. Similar conclusions are drawn by other studies, which only approximate socioeconomic level through educational level and social class for Spain [34, 7], Canada [35] and Norway [33].

After the onset of the financial crisis in 2011, the previous result was modified only by individuals in the 3rd quartile being relatively less likely to use these types of services than individuals in the 1st income quartile (more specifically, 11% less), with no significant differences for the rest of the quartiles. It is possible that part of this lower relative probability for individuals in the 3rd quartile is due to the fact that this group have found themselves more affected by the reduction in the number of appointments to ask for sick leave (given the regulatory changes that occurred) or by the consolidation of the electronic prescription. When we observe the pattern of utilisation of privately funded general medical services by income levels, if in 2006 individuals in the 4th income quartile had a five-times greater relative probability of using these services than individuals in the 1st quartile, this effect had disappeared by 2011, which suggests that the crisis has caused higher-income households to use private family doctor services relatively less. Therefore, it may be that there has been a transfer in the utilisation of the corresponding public services on the part of the 4th quartile (which would explain why they do not join the 3rd quartile in their relatively less utilisation of the public family doctor services).

### Specialist medical services

Neither were there any significant differences in the utilisation of public appointments by income levels before the crisis. These results are consistent with some previous studies where no significant differences were detected in utilisation by income levels [9–11] or by socioeconomic levels [15]. Most previous studies, however, did find that income was positively related to greater utilisation of specialist healthcare services, both in Spain [8, 12] and internationally [2, 31, 33, 35]. Logically, this relation was also found by studies that made international comparisons and which took privately funded appointments into account, as well as publicly funded ones [1, 30, 31, 36]. Part of the divergence between the results of these last studies and those obtained by our research could be explained by the different adjustment for need carried out in the former (which used different variables like self-evaluation of one’s state of health or the existence of chronic diseases), as evidenced in Agerholm et al. [15] for outpatient visits in Sweden. In any case, in this research our main interest was comparing the situation before and after the financial crisis and so the design of the study and the selection of variables (as well as the health need variables) were conditioned by this objective.

Having said that, after the crisis began individuals from the 2nd quartile were once again less likely to see a specialist (14% less likely than those in the 1st quartile), thus generating a certain regressivity in the distribution of these public services. On the other hand, as for private family doctor appointments, for private specialist appointments there was a clear gradient before the crisis (individuals in the 3rd and 4th quartiles had a 3.5 and 4.5 times greater relative propensity than individuals in the 1st quartile to see a private medical specialist). This effect disappeared after the crisis began, lending credence to the idea that the financial crisis caused a decrease in the private utilisation of these types of health services among higher-income households, who now asked for public health services, thus contributing to the appearance of this gradient in public health service utilisation that emerged after the onset of the crisis and was detrimental to individuals in the 2nd income quartile.

### Hospitalisations

In 2006, there was a steep gradient in publicly financed hospitalisations, such that belonging to the 2nd, 3rd or 4th income quartiles was associated with a monotonous decrease in the likelihood of being hospitalised (i.e. a lower relative probability of 28%, 58% and 60% respectively, in comparison to individuals in the 1st income quartile). This result contrasts with the findings of previous studies like those of Abásolo et al. [11, 12] for Spain and Morris et al. [13] for the United Kingdom, where income had a positive effect on the probability of being hospitalised in a public hospital. As explained earlier, this difference could be due to the fact that the previous studies used other health variables to adjust for both health need and socioeconomic level (educational level and social class). In fact, our results are along the same lines as those of Regidor et al. [8], who adjusted only for age and sex, and of Regidor et al. [9], who also adjusted for the number of chronic diseases individuals suffered from.

However, in 2011 after the crisis began, this steep gradient disappeared completely and there were no significant differences by income quartiles in the relative probability of being hospitalised. On the other hand, bear in mind that for privately funded hospitalisations no significant association with household incomes was detected either before or after the onset of the crisis. Despite this, it must be remembered that the lower frequency (and, therefore, the smaller sample size) of the identification of this utilisation, makes the conclusions concerning private funding less robust than those concerning public funding.

### Utilisation of emergency services

Regarding the utilisation of public emergency services, there is less previous evidence in the available literature. For Spain, Urbanos 2001 [17] found no significant differences in the utilisation of emergency services either by educational level or by social class - only the highest social class used these services less intensely than the rest of the population. Our results of the estimations for 2006 indicate that there is a clear gradient in favour of the low-income groups: more specifically, individuals in the 2nd, 3rd and 4th quartiles had a lower relative probability of using the emergency services (33%, 50% and 53% less respectively) than individuals in the 1st income quartile. This marked gradient by income level not only continued after the crisis began, but by 2011 was more accentuated because by then the relative probabilities previously mentioned had gone up to 54%, 55% and 66% less, respectively. In the case of the private emergency services, in 2006 only those in the 2nd income quartile used these services less, an effect which had disappeared by 2011 and from which no clear effect can be detected on the potentiation of the pro-poor distribution that emergencies in the public health service underwent after the onset of the crisis.

### Medication

Previous studies such as Nordin et al. [37] have shown that there is an education gradient (attributable to the behaviour of doctors), although not by income level, in the use of medication. Our results for 2006 show a steeper income gradient for medication use for the poorest: individuals in the 3rd and 4th quartiles had a lower relative probability (12% and 13% less, respectively) of taking medication than individuals in the 1st income quartile, an effect that was detected once adjustment was made for age, sex and mental health status. Even without adjustment, individuals in the 2nd quartile were relatively less likely to take medication. However, after the crisis began, by 2011 there was a reduction in the use of medication, and distribution in favour of the lowest income groups had slipped towards the middle-income groups: then, it was individuals from the 2nd and 3rd quartiles that had an increased relative probability of 10% and 7%, respectively, of taking medication, than individuals in the 1st income quartile; this time, it was adjustment for need (age, sex and mental health) and double medical provision that allowed us to see that medication use for the 4th quartile was no longer significantly different from the 1st quartile. However, we must take into account the fact that, in the case of medication, the analysis for their public funding could not be carried out (total funding), so it was difficult to make hypotheses similar to the proposals made for the rest of the health services. It must also be noted that the change in the co-payment of outpatient medication produced by the RDL 16/2012, which came into effect on 1st July 2012, had not yet come into effect at the time of the analysis.

### The effect of other factors (need indicators and double medical cover)

We assume that the possible change that could occur after the onset of the crisis in the effect of health need on the use of health services by income level would be reflected in the variables that we consider: age, sex and mental health. We decided not to include self-perception of the state of one’s health as we believe this to be a variable that is very much influenced by a situation that is not always well-defined in each period. Neither did we consider the rest of the chronic diseases because we assumed that the crisis has not created more barriers to health service utilisation for these types of diseases. In the cases of specialist healthcare services, hospitalisations and medication with public funding, we believe that adjustment for need plays a secondary role as we are dealing with services and goods that must be ordered by healthcare professionals (who are qualified to decide whether or not the patient has a health need). This is not the case with either publicly funded general medical and emergency services or with all privately funded services, as for these services patient choice weighs heaviest; they are services for which we trust that the variables of need used in our study are sufficient, given the aims of this research. It may be that part of the differences between the results of our research and those of previous studies are due to this minor adjustment for proxy variables of the health of the individuals. In any case, we must emphasise that the aim of our study is not so much to analyse inequity by income in the distribution of public health services, but to compare the situation before and after the onset of the economic crisis regarding inequalities in utilisation by income levels, once they have been adjusted by the variables of need, whose effects we anticipate could have changed between 2006 and 2011.

Special mention must be made of the behaviour of the demand for private health insurance (i.e. double medical cover) with the onset of the crisis: First, we are concerned with the association of the crisis and the demand for private health insurance; and second, with the relationship between the crisis and the propensity of those with double health cover to use health services (whether public and private) more or less intensely. As regards the first relationship, after the crisis began there was a significant increase (7.2%) in the demand for private health insurance (in 2006, 12.5% of those interviewed had private cover, a percentage that rose to 13.4% in 2011) (Table 2). Contrary to what could be surmised, this increase was due to the marked rise in demand for private health insurance among individuals in the 1st quartile, as in 2006 only 10% of this group had private insurance and this figure had increased to 15% by 2011 (a rise of 54%). In the case of the 2nd and 3rd quartiles, a decrease in private health insurance was registered (6.7% and 7.2% less than in 2006, respectively), while there was no significant change for the 4th quartile (Table 3). To the extent that this increased demand for private insurance among the poorest is due to an ‘expulsion’ effect brought about by a greater use of public health services by higher income groups in particular, it is yet another manifestation of a more regressive distribution of public health services since the onset of the crisis.

Regarding the second relationship, the financial crisis has been accompanied by an interesting result in view of the propensity of those with double health cover to use health services. In relation to specialist public services – the main focus of this research – the relative propensity to use these services reduces when there is private health insurance, as was to be expected and was evidenced previously [10, 38]. More specifically, in 2006 this propensity reduced by 14% with respect to those without double health cover, a relation that was maintained after the onset of the crisis, although slightly less intensely (11% less). However, the greater propensity on the part of individuals with private health cover to use private specialist services in 2006 (double the relative probability of utilisation) disappeared in 2011. If our adjustment by health need is correct, and there have been no major changes in this sense (and no major changes in the co-payment system of insurance companies), it would seem that the crisis has also created greater obstacles to the private use of services by those that are insured (as the decrease in the lower propensity to see a private specialist mentioned Spreviously is not enough to justify this drastic reduction in the utilisation of private specialist appointments). The overall effect on all services (both public and private) shows that if in 2006 the relative probability of seeing a specialist was 27% greater for those with private health insurance, by 2011 there were no longer any significant differences between those that had private insurance and those that did not. Regarding medication, in 2006 there was a slightly higher propensity to take medication among individuals with private medical cover, but this had also disappeared by 2011. Finally, regarding hospitalisations and emergencies, the main differences occurred in the realm of private healthcare. In 2006, those with private medical cover had a 33% greater relative probability of being hospitalised in a private hospital and a 13% greater probability of using a private emergency service, differences that disappeared with the onset of the crisis, with no significant effect either before or after the crisis as these two services are publicly financed.

## Conclusions

Within the Spanish National Health System, the onset of the financial crisis has been accompanied by a decrease in family doctor appointments and hospitalisations and an increase in appointments with specialists, while utilisation of the emergency services has remained constant. Furthermore, these changes have not been neutral with respect to the different income groups. With respect to public specialist services, since the crisis began there has been an increase in the use by individuals in all of the income quartiles, but once adjustment is made for health need it can be seen that before the crisis there was no significant difference in utilisation for the different income quartiles (contrary to what was observed in general in the literature for previous periods) and that after the onset of the crisis, individuals in the 2nd quartile had a lower relative probability of seeing a specialist, thus introducing a certain regressivity into the utilisation of these public health services. On the other hand, in the case of private specialist appointments, even though there was a certain increase in utilisation for all income groups, the steep gradient in favour of higher income groups disappeared once the crisis began, which would indicate a change from private to public in the propensity to use these types of health services, which would help to explain the previously mentioned regressivity.

Public family doctor appointments have a specific profile. After the crisis began, primary healthcare services utilisation underwent a decrease, which could be the result of less demand for appointments to ask for sick leave or because of the consolidation of the electronic prescription, as noted in the introduction. The adjustment for need suggests that after a neutral distribution with respect to income for these types of public health services, the crisis only seems to be associated with a lower propensity to use these services by individuals in the 3rd income quartile. When we look at what has happened with privately funded family doctor services, if in 2006 individuals in the 4th income quartile had a relative probability of utilisation five times greater than those in the 1st income quartile, by 2011 this effect had disappeared, which could help to explain that the new distribution after the onset of the crisis only affects individuals in the 3rd (and not the 4th) income quartile.

In the case of public hospitalisations, after the onset of the crisis there has been both a decrease in the utilisation of these types of services and their redistribution: the pro-poor distribution that existed pre-crisis later disappeared, which would indicate that either higher income groups are using public hospital services more intensely or that lower income groups are tending to use them less intensely. A very different result was produced in the case of emergencies: use of these services did not increase during the period under study, but the pro-poor distribution that existed pre-crisis was even more accentuated afterwards. With respect to medication, after the crisis began there was a decrease in the use of medication and the pre-crisis distribution in favour of lower income groups slipped towards middle income groups, even though in this case there is mixed funding (public and private) and not only for comprehensive universal cover and uses with a prescription.

Last, with respect to double medical cover (private health insurance as well as public healthcare), the results of our analysis indicate that the crisis has brought about an increase in the demand for private health insurance, an increase fostered by the demand from individuals in the 1st income quartile (but not so for the 2nd or the 3rd quartile, amongst whom less private health insurance was purchased, and not for the higher quartile amongst whom demand remained constant). Furthermore, the results suggest that the crisis has also been associated with a change in the pattern of private healthcare utilisation among those with double medical cover, a new pattern that does not seem to be explained solely by the substitution of these services by publicly funded ones. Unless the profile of private healthcare users has changed in a way that could justify this finding (something which we cannot know from the information available to us in this study), it may be that insurance companies, who are also adversely affected by the crisis, have erected more barriers against moral hazard and have reduced user access, especially to specialist and emergency care services.

The results of this research show that universality in public healthcare provision has not prevented the financial crisis from affecting some income groups more than others when it comes to using public health services. Neither should we be surprised by the increased relative utilisation of the public health system by the richest and their abandonment of the private system for high-cost services, or a larger proportion of low-income individuals with private health insurance or, of course, by the variation in health need relative to new socioeconomic conditions. In the case of Spain, following the onset of the recent crisis there has been a decrease in the utilisation of specialist and hospitalisation services by the lower income groups who, on the other hand, have benefitted in terms of emergency treatment and family doctor appointments.

These results suggest that health authorities should better monitor the effects of the financial crisis on access to public health services for all income levels, especially if a worsening of the crisis brings about negative effects on the health of citizens. Furthermore, our results suggest that the two inequalities that emerge from the crisis must be dealt with differently: redressing the inequality that adversely affects the poorest requires measures to modify guidelines for healthcare professionals (demand for specialist services and hospitalisation initiated by healthcare professionals), while redressing the inequality that benefits the poorest requires measures that affect the incentives of the patients themselves (demand for emergency services and family doctor appointments initiated by patients) and in particular the cost of the opportunity to access these services.

## Research highlights

- The recession has been more detrimental to low-income groups in specialist appointments.
- The same was the case for hospitalisations,
- The recession had worked to their advantage in the cases of emergency services.
- This was also the case for family doctor appointments.

## Abbreviations

GHQ-12: Goldberg’s mental health index
INLA: Integrated Nested Laplace Approximation
PC priors: Penalising complexity priors
SNHS: Spanish National Health Survey

## Footnotes

1 Which amounted to 76.77% of the individuals who used primary health care in SNHS-2006 and 79.13% in SNHS-2011-2012, 82.48% in SNHS-2006 and 82.08% in SNHS-2011-2012 for specialized health care, 67.95% in SNHS-2006 and 65.87% in SNHS-2011-2012 for emergency room services, and for hospitalizations 87.57% in SNHS-2006 and 78.56% in SNHS-2011-2012.
