# Correction to: Financial crisis and income-related inequalities in the universal provision of a public service: the case of healthcare in Spain

**DOI:** 10.1186/s12939-019-0972-8

**Published:** 2019-05-09

**Authors:** Ignacio Abásolo, Marc Saez, Guillem López-Casasnovas

**Affiliations:** 1 Department of Applied Economics and Quantitative Methods, University of La Laguna, La Laguna, Spain; 2 University Institute of Regional Development, University of La Laguna, La Laguna, Spain; 3 Research Group on Statistics, Econometrics and Health (GRECS) University of Girona, Carrer de la Universitat de Girona 10 Campus de Montilivi, 17003 Girona, Spain; 4 CIBER of Epidemiology and Public Health (CIBERESP), Madrid, Spain; 5 Center for Research in Health and Economics (CRES), University Pompeu Fabra, Barcelona, Spain; 6 Department of Economics and Business, University Pompeu Fabra, Barcelona, Spain; 7 Barcelona Graduate School (BSGE), University Pompeu Fabra, Barcelona, Spain

**Correction to: Int J Equity Health (2017) 16:134**

**https://doi.org/DOI 10.1186/s12939-017-0630-y**

Following publication of the original article [1], the autor reported 5 references should be indicated in Spanish. The correct references can be found below:

4. Calonge S, Manresa A. Consecuencias distributivas y de equidad de las políticas de gasto y financiación de la sanidad. Moneda y Crédito. 1997; 204: 13–66. Google Scholar
5. Ortiz F, Abásolo I, Jiménez V. Sanidad Pública y Distribución de la Renta en España. En Políticas de Bienestar y Desempleo (coord. José M. Maravall Herrero). Madrid: Fundación Argentaria; 1999. Google Scholar
10. González-Álvarez ML, Clavero-Barranquero A. Análisis de las desigualdades socioeconómicas en la utilización de asistencia sanitaria mediante modelos dinámicos. Hacienda Pública Española. 2008; 186(3): 9–42. Google Scholar
11. Abásolo I, Pinilla J, Negrín M. Equidad en la utilización de servicios sanitarios públicos por Comunidades Autónomas en España: un análisis multinivel. Hacienda Pública Española. 2008;187(4):87–106. Google Scholar
12. Abásolo I, Negrín M, Pinilla J. Utilización y tiempos de espera: dos vertientes inseparables del análisis de la equidad en el acceso al sistema sanitario público. Hacienda Pública Española. 2014; 208: 11–38. Google Scholar.
